# Atypical Antiglomerular Basement Membrane Disease in a Pregnant Patient with Systemic Lupus Erythematosus

**DOI:** 10.1155/2023/6963543

**Published:** 2023-11-11

**Authors:** Areerat Ounhasuttiyanon, Ngoentra Tantranont, Thatsaphan Srithongkul

**Affiliations:** ^1^Division of Nephrology, Department of Medicine, Faculty of Medicine, Siriraj Hospital, Mahidol University, Salaya, Thailand; ^2^Department of Pathology, Faculty of Medicine, Siriraj Hospital, Mahidol University, Salaya, Thailand

## Abstract

Antiglomerular basement membrane disease (anti-GBM) is an unusual cause of glomerulonephritis. Patients usually present with rapidly progressive glomerulonephritis with or without pulmonary hemorrhage. The diagnosis is based on linear deposits of IgG along the GBM and the presence of anti-GBM antibodies. However, cases with atypical anti-GBM disease in which an anti-GBM antibody was not detected have been reported. We report a 29-year-old pregnant woman with underlying systemic lupus erythematosus (SLE) who presented with severe glomerulonephritis due to atypical antiglomerular basement membrane disease. She was initially diagnosed with active lupus nephritis and her renal function gradually worsened after steroid treatment, so the pregnancy was terminated due to the high maternal and fetal risks. A kidney biopsy showed linear capillary wall staining with fibrous crescents without endocapillary proliferation. The anti-GBM antibody showed negative results two times, so she was diagnosed with atypical anti-GBM disease. Treatment began with intravenous pulse methylprednisolone and continued with mycophenolate mofetil and prednisolone. Due to the intolerability of side effects, the treatment regimen was subsequently changed to intravenous cyclophosphamide. Although she had a significant improvement in clinical edema, serum albumin, and hematuria, her renal function gradually decreased during the 12 months of treatment. A review of the literature showed that the atypical anti-GBM is less aggressive than the typical anti-GBM disease. However, several patients had persistent renal dysfunction and 20–30% of patients had progression to ERSD. To the best of our knowledge, this is the first case of atypical anti-GBM disease in pregnant patients with suspected SLE reported in the literature.

## 1. Introduction

Antiglomerular basement membrane (anti-GBM) disease is a rare small vessel vasculitis caused by autoantibodies that target type 4 collagen in the basement membrane. Data on true incidence are lacking, with an estimated incidence of 0.5–1.8 per million population/year in both Caucasians and Asian populations [1]. The incident was observed at a bimodal age, with a peak incidence at 30 and 60–70 years [2]. Most patients typically present with rapidly progressive glomerulonephritis (RPGN), and pulmonary involvement was found in the form of alveolar hemorrhage (Goodpasture's disease) in 40–60% of patients [2]. The diagnosis is based on the presence of anti-GBM antibody and characteristic histological findings with diffuse crescentic glomerulonephritis and linear IgG staining on biopsy tissues. Anti-GBM disease is one of the most aggressive forms of acute glomerulonephritis. The 1-year patient and renal survival rates have been reported to be 73% and 25%, respectively [3], and the 5-year patient and renal survival rates were 83% and 34%, respectively [4]. A previous study correlated the presentation with oliguria or severe renal dysfunction and the proportion of crescentic glomeruli with poor renal outcomes [2]. Some patients developed end-stage renal disease even with aggressive immunosuppressive treatment, including plasmapheresis. In addition to the classic anti-GBM disease, atypical presentations of the anti-GBM disease have been reported. The diagnosis was based on the linear accumulation of immunoglobulins along the GBM on kidney biopsy, while an anti-GBM antibody was not detected. According to the case report and case series, the clinical presentation, pathology, and prognosis of atypical anti-GBM disease are different from typical anti-GBM disease. Here, we report a case of atypical anti-GBM disease in a pregnant woman with suspected systemic lupus erythematosus (SLE).

## 2. Case Presentation

A 29-year-old Thai woman with a significant medical history of suspected SLE was diagnosed in 2018 due to a criteria diagnosis of arthritis and positive antinuclear antibody (ANA) 1 : 1280 in fine speckle and homogeneous pattern, thrombocytopenia. She was clinically stable and had never had renal involvement before. Her current medications were prednisolone 5 mg on an alternate day and hydroxychloroquine 200 mg per day. She presented to the outpatient department with a 2-month history of progressive bilateral leg edema and gained 2 kilograms. She reported dark, foamy urine and oliguria for a few weeks before. She denied fever, joint pain, skin rash, oral ulcer, thinning hair, shortness of breath, cough, chest pain, abdominal pain, vomiting, diarrhea, or dysuria. She had no history of alcohol, smoking, or drug abuse. She did not use any contraceptives and her period had been missed for two months.

On physical examination, her blood pressure was 129/72 mmHg. The patient was alert, oriented, and in no acute distress. The rest of the examination was notable for mild pale conjunctiva and bilateral pitting edema in the legs 3+ without other significant findings.

Laboratory investigation showed hemoglobin levels of 8.8 g/dL, hematocrit of 25.6%, white blood cell count of 12370/*μ*L, platelet count of 242,000/*μ*L, blood urea nitrogen levels of 24.6 mg/dL, creatinine (Cr) levels of 1.83 mg/dL (her baseline Cr was 0.94 mg/dl 1 year ago), and albumin levels of 2.9 mg/dL. Urinalysis showed 100–200 red blood cells/HPF, 1-2 white blood cells/HPF, and a urine protein creatinine ratio (UPCR) of 7 g/g Cr. Further evaluation revealed that the patient had normal complement levels (C3 and C4), positive fine speckle pattern for ANA (titer 1 : 1000), negative for anti-dsDNA, negative for anti-sm, negative for antiphospholipid antibody, and negative for anti-GBM antibody after two repeated tests using enzyme-linked immunosorbent assay (ELISA). The serologies for hepatitis B and C were negative. The renal ultrasound showed increased parenchymal echogenicity of both kidneys with normal size and cortical thickness. Her urine pregnancy test was positive and pelvic ultrasound confirmed a nine-week-size intrauterine pregnancy.

As a result, she was first diagnosed with pregnancy with suspected active SLE and lupus nephritis. The treatment started with prednisolone 40 mg daily (1MKD). A gynecologist was also consulted for a pregnancy care plan. She was advised to terminate the pregnancy due to the high maternal and fetal risk from her medical condition. However, the patient and her husband preferred to follow-up on her renal function and try medical treatment first. On a follow-up visit, the patient had progressive lower limb edema with difficulty in walking and gained 8 kilograms in 1 month. Her serum Cr was stable at around 1.8 mg/dL. After a lengthy discussion, the patient and her husband decided to terminate the pregnancy at 13 weeks of gestational age. She underwent fractional and curettage, and a kidney biopsy was scheduled one week later.

**Figure 1:**
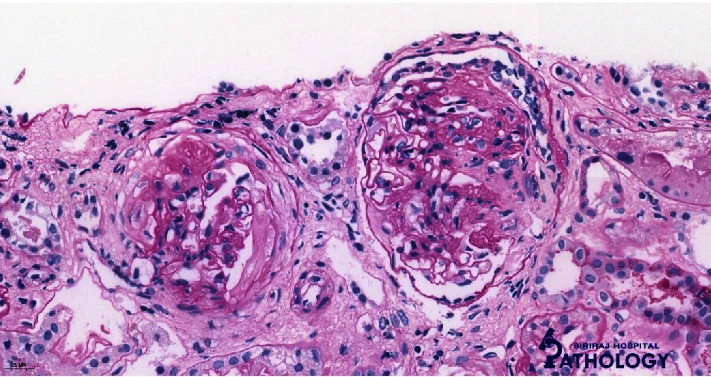
PAS staining showing glomeruli with segmental sclerosis, podocyte hyperplasia, and fibrous crescent.

**Figure 2:**
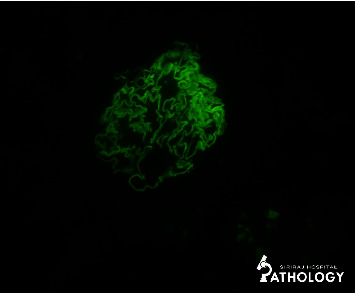
Immunofluorescence staining for immunoglobulin G showing a diffuse strong linear staining pattern.

A light microscopic study revealed a total of 22 (6 globally sclerotic, 14 segmentally sclerotic, and 2 normal glomeruli) but 2 normal glomeruli were not mentioned, so there was no mismatch in the number. Five glomeruli had a fibrous crescent (Figure 1). Podocyte hyperplasia was observed in several glomeruli. The mesangium showed no increase in cell or matrix. There was no abnormal thickening of the capillary wall, endocapillary proliferation, fibrinoid necrosis, or thrombosis. Furthermore, there was mild tubular atrophy (5%) and interstitial fibrosis (5%) with interstitial mononuclear cell infiltration. Patchy foci of acute tubular injury were also observed. Immunofluorescent studies showed a glomerulus with bright linear GBM staining for IgG (2+) (Figure 2), lambda (2+), and kappa (1+). In addition, there is a segmental granular capillary wall staining for IgM (1+) and C1q (trace) and negative staining for C3. Unfortunately, we could not perform a subclass for IgG in our center and there were not enough glomeruli for the electron microscopy (EM) study. The kidney pathology was compatible with the anti-GBM disease. Due to the negative result of the anti-GBM antibody, her definite diagnosis was atypical anti-GBM disease.

Her serum creatinine peaked at 2.47 mg/dl during admission. After the diagnosis of atypical anti-GBM was established, the treatment was started with intravenous methylprednisolone 500 mg daily for three days and planned to follow by prednisolone 40 mg daily. However, the patients developed endometritis, so the prednisolone dose was decreased to 15 mg daily. Piperacillin/tazobactam was started and changed to oral amoxicillin/clavulanate. She responded well to antibiotics and her clinical condition improved, so mycophenolate mofetil (MMF) was started at 1000 mg daily combined with oral prednisolone of 20 mg and continued until discharge. Her creatinine before discharge remained stable.

**Figure 3:**
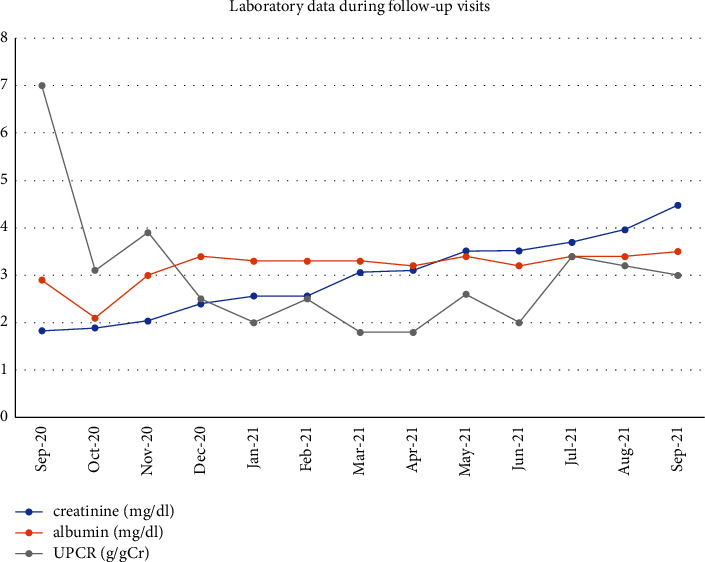
Laboratory data during patient follow-up visits.

During a follow-up visit, her serum creatinine remained stable at 2.0–2.5 mg/dl and the laboratory data are shown in Figure 3. MMF was up titrated to 1500 mg/d, but the patient had gastrointestinal side effects of nausea and vomiting. As a result, MMF was discontinued after two months, and the patient was treated with a 6-month course of intravenous cyclophosphamide (cumulative dose of 5.2 grams) and oral prednisolone. Her creatinine gradually increased and reached 3.5 mg/dL after a complete course of intravenous cyclophosphamide. Azathioprine 50 mg daily and oral prednisolone 10 mg daily were continued thereafter. At 12 months after starting treatment, the patient's creatinine was 4.48 mg/dL (eGFR 12.45 ml/min/1.73 m^2^). Her UPCR was 2-3 g/g Cr with no hematuria and serum albumin of 3.2–3.9 g/dl.

## 3. Discussion

An atypical anti-GBM disease is a unique form of anti-GBM disease in which no anti-GBM antibody is detected in serum. Patients have different clinical characteristics from those with the classic anti-GBM disease. Here, we report a case of a 29-year-old pregnant woman with underlying systemic lupus erythematosus (SLE) with severe glomerulonephritis from atypical anti-GBM disease.

To our knowledge, this is the first case of atypical anti-GBM disease in pregnant patients with suspected systemic lupus erythematosus reported in the literature, and to date, there are only approximately ten case reports of anti-GBM antibody glomerulonephritis in pregnant patients [5–10], and only two of them are negative against anti-GBM antibodies [8, 9]. Al-Harbi et al. reported a 30-year-old woman with acute renal failure at 28 weeks of gestation, whose repeat anti-GBM antibody test in a serum sample taken after delivery was positive [8]. Moreover, Deubner et al. also described a case of anti-GBM antibody glomerulonephritis in a 21-year-old woman at 30 weeks of gestation, in which during pregnancy anti-GBM was detected with a low titer, and the level increased after delivery, consistent with patient kidney failure that worsened after delivery [7]. In another case report of a 28-year-old woman who established anti-GBM antibody glomerulonephritis before conception, the anti-GBM antibody became negative during pregnancy [6]. To explain these findings, Deubner et al. postulated that the placenta may have served as an adsorptive surface for the autoantibody, and there was some evidence that binding of anti-GBM antibodies to placental membranes can occur [7]. This hypothesis may explain the decrease in anti-GBM level in maternal serum and the improvement of its manifestations antepartum, and after the placenta removal, more antibodies were available to bind to GBM and thus, an increased decline in renal function postpartum. Our case also demonstrated atypical anti-GBM disease in a pregnant patient, in which a negative level of GBM antibody may be explained by a low level of circulating antibodies. However, this could not be confirmed because no postpartum serum samples were collected for the anti-GBM antibody level.

Our patient was diagnosed with SLE with 2019 EULAR/ACR classification criteria with a score of 10 due to ANA +1 : 1000, a history of thrombocytopenia, and arthritis. Although there are some reports of SLE and anti-GBM disease, there is no report of SLE and atypical anti-GBM disease. All of them have positive anti-GBM antibodies, but only some of them have linear IgG deposits along the GBM. In a retrospective study from China of 157 hospitalized patients with SLE, anti-GBM was detected in 14 (8.9%) patients and all patients developed LN, but only 9 of 14 had typical linear immunoglobulin G (IgG) and C3 deposition in the GBM [11]. It was hypothesized that the presence of lupus nephritis exposed GBM antigens and promoted the production of the anti-GBM antibody. There is also a reported case of identical twins who had anti-GBM nephritis and SLE, respectively, providing clinical evidence to support that anti-GBM nephritis and lupus may share common genetic risk factors. Studies found that HLA-DRB1*∗*1501 has been associated with susceptibility to both anti-GBM disease and SLE [12]. However, in the largest anti-GBM antibody study conducted in patients with and without LN with SLE in Europe, anti-GBM antibodies were not detected in serum from Caucasian patients with SLE, even in cases of renal involvement [13]. These may be explained by different genetic backgrounds in the population between studies. In our case, unlike previously reported cases, there was no evidence of lupus nephritis, and there was a negative anti-GBM antibody in the serum, and there was no extrarenal manifestation of active SLE flares with normal complement and anti-dsDNA at the time of progressive renal dysfunction, so we cannot conclude the association between SLE and atypical anti-GBM disease in this patient. However, the negative anti-GBM may be explained by pregnancy as previously described.

Anti-GBM disease in pregnancy is associated with poor maternal and fetal outcomes, as well as in the general population, but the prognosis of atypical anti-GBM disease was better than typical anti-GBM disease, and most patients responded well to mycophenolate or cyclophosphamide. However, the clinical course of atypical anti-GBM in pregnancy is still unknown because only a few cases have been reported. In three reported cases, including our cases, two patients were dialysis-dependent and one patient fully recovered.

However, the clinical condition of our patient did not improve after taking mycophenolate mofetil and cyclophosphamide. Subsequently, her kidney function decreased. The poor response may be due to the nature of the disease, 23–32% in the case series of atypical anti-GBM disease developed ERSD [14–16], or may be the result of her intolerability to the titration of MMF dose due to gastrointestinal side effects.

## 4. Conclusion

The clinical characteristics and findings on pathological examinations of patients with atypical anti-GBM disease were diverse, with milder disease severity and more favorable renal outcomes compared to the classical anti-GBM disease. However, many of those with atypical anti-GBM disease have persistent renal dysfunction and 20–30% of patients have progression to ERSD. This highlighted the early diagnosis of atypical anti-GBM by kidney biopsy due to negative serum anti-GBM and prompt treatment should be initiated because not all patients have a favorable prognosis. However, treatment options need further exploration in clinical trials due to limited data on patient outcomes.

## Data Availability

The data for this case can be provided upon reasonable request to the corresponding author.

## References

[1] Tang W., McDonald S. P., Hawley C. M. (2013). Anti-glomerular basement membrane antibody disease is an uncommon cause of end-stage renal disease. *Kidney International*.

[2] McAdoo S. P., Pusey C. D. (2017). Anti-Glomerular basement membrane disease. *Clinical Journal of the American Society of Nephrology*.

[3] Chen M., Cui Z., Zhao M. H. (2010). ANCA-associated vasculitis and anti-GBM disease: the experience in China. *Nephrology Dialysis Transplantation*.

[4] Van Daalen E. E., Jennette J. C., McAdoo S. P. (2018). Predicting outcome in patients with anti-GBM glomerulonephritis. *Clinical Journal of the American Society of Nephrology*.

[5] Thomson B., Joseph G., Clark W. F. (2014). Maternal, pregnancy and fetal outcomes in de novo anti-glomerular basement membrane antibody disease in pregnancy: a systematic review. *Clinical Kidney Journal*.

[6] Yankowitz J., Kuller J. A., Thomas R. L. (1992). Pregnancy complicated by Goodpasture syndrome. *Obstetrics & Gynecology*.

[7] Deubner H., Wagnild J. P., Wener M. H., Alpers C. E. (1995). Glomerulonephritis with anti-glomerular basement membrane antibody during pregnancy: potential role of the placenta in amelioration of disease. *American Journal of Kidney Diseases*.

[8] Al-Harbi A., Malik G. H., Al-Mohaya S. A., Akhtar M. (2003). Anti-glomerular basement membrane antibody disease presenting as acute renal failure during pregnancy. *Saudi J Kidney Dis Transpl*.

[9] Sprenger-Mähr H., Zitt E., Soleiman A., Lhotta K. (2019). Successful pregnancy in a patient with pulmonary renal syndrome double-positive for anti-GBM antibodies and p-ANCA. *Clinical Nephrology*.

[10] Kai H., Usui J., Tawara T. (2021). Anti-glomerular basement membrane glomerulonephritis during the first trimester of pregnancy. *Internal Medicine*.

[11] Li C. H., Li Y. C., Xu P. S., Hu X., Wang C. Y., Zou G. L. (2006). Clinical significance of anti-glomerular basement membrane antibodies in a cohort of Chinese patients with lupus nephritis. *Scandinavian Journal of Rheumatology*.

[12] Liu X., Wu Y., Yang Y. (2013). Identical twins:one with anti-glomerular basement membrane glomerulonephritis,the other with systemic lupus erythematosus. *BMC Nephrology*.

[13] Bourse Chalvon N., Orquevaux P., Giusti D. (2020). Absence of anti-glomerular basement membrane antibodies in 200 patients with systemic lupus erythematosus with or without lupus nephritis: results of the GOODLUPUS study. *Frontiers in Immunology*.

[14] Shen C., Jia X., Cui Z., Yu X., Zhao M. (2020). Clinical-pathological features and outcome of atypical anti-glomerular basement membrane disease in a large single cohort. *Frontiers in Immunology*.

[15] Nasr S. H., Collins A. B., Alexander M. P. (2016). The clinicopathologic characteristics and outcome of atypical anti-glomerular basement membrane nephritis. *Kidney International*.

[16] Liang D., Liang S., Xu F. (2019). Clinicopathological features and outcome of antibody-negative anti-glomerular basement membrane disease. *Journal of Clinical Pathology*.

